# Valorization of Sugarcane Bagasse Ash and Steel Slag in Concrete: Experimental Evaluation of Mix Performance and Structural Properties

**DOI:** 10.3390/ma19122472

**Published:** 2026-06-09

**Authors:** Bane Ibsa Tola, Zakarias Gebreyes Eticha, Jemal Jibril Muhammed, Jose Henriques

**Affiliations:** 1 Faculty of Material Science and Engineering, Jimma Institute of Technology, Jimma University, Jimma 378, Ethiopia; baneibsatola@gmail.com; 2 Industrial Parks Development Corporation-Industrial Projects Service, Addis Ababa 2569, Ethiopia; hgoldnet@gmail.com; 3 Faculty of Civil and Environmental Engineering, Jimma Institute of Technology, Jimma University, Jimma 378, Ethiopia; jibril.mohammed@ju.edu.et; 4 Construction Engineering Research Group (CERG), Faculty of Engineering Technology, Hasselt University, 3500 Hasselt, Belgium

**Keywords:** sugarcane bagasse ash, steel slag, sustainable concrete, cement replacement, mechanical performance, mix optimization, industrial waste valorization, compressive strength, structural concrete

## Abstract

**Highlights:**

- Sugarcane bagasse ash (BAC) and steel slag (SS) used as cement and sand replacements.
- Strength development evaluated at 7 and 28 days for reference and alternative concretes.
- Up to 15% BAC, 25% SSC, and 50% SSS maintained structural strength performance.
- Mix (15% BAC + 50% SSS) meets strength criteria with balanced performance and material substitution.
- Confirms feasibility of industrial waste valorization for sustainable concrete.

**Abstract:**

This study investigates the use of sugarcane bagasse ash (SCBA) and steel slag (SS) as partial replacements for cement and natural river sand in concrete, with the objective of identifying replacement levels that maintain structural performance while reducing the consumption of conventional materials. An experimental program was conducted to evaluate the unit weight, compressive strength, and splitting tensile strength of concrete containing SCBA and SS in individual and combined replacement systems. The results showed that the incorporation of SCBA reduced concrete density, whereas SS increased unit weight due to its higher specific gravity. At 28 days, compressive strength ranged from 13.09 to 38.10 MPa, while splitting tensile strength varied between 1.81 and 4.74 MPa, depending on the replacement level and combination of materials. Among the investigated mixtures, the concrete containing 15% SCBA and 50% SS exhibited the most favourable overall performance, achieving the target compressive strength of 25 MPa required for structural applications while maintaining acceptable tensile strength. In contrast, higher replacement levels resulted in strength reductions attributed to cement dilution, increased porosity, and the delayed pozzolanic reactivity of SCBA. Overall, the findings demonstrate that appropriately proportioned SCBA and SS can be successfully incorporated into concrete without compromising structural performance. The optimal mixture provides an effective balance between mechanical performance and the utilization of alternative raw materials, highlighting the potential of these industrial by-products to support more sustainable concrete production.

## 1. Introduction

The rapid expansion of the global construction sector has resulted in substantial consumption of natural resources and increased greenhouse gas (GHG) emissions. Conventional construction materials such as cement and concrete are produced through energy-intensive processes that rely heavily on fossil fuels. Cement manufacturing alone accounts for approximately 8% of global anthropogenic CO_2_ emissions due to clinker production and calcination processes [1]. In addition to carbon emissions, cement production contributes to land degradation, air pollution, and resource depletion through raw material extraction and high-temperature kiln operations [2].

Rapid urbanization and housing demand, particularly in developing countries, are expected to further intensify environmental pressures associated with construction activities. It is projected that construction-related GHG emissions could double by 2050 if conventional materials and practices persist [3]. In Ethiopia, where urban growth and infrastructure development are central to national economic strategy, the construction sector has expanded significantly in recent years [4]. However, the industry remains highly dependent on conventional materials, many of which are energy-intensive or imported, thereby increasing both environmental and economic burdens.

Historically, traditional Ethiopian housing relied on locally available earth-based materials, such as “Chika bet” (mud, straw (grass) and wood), which offered low embodied energy but suffered from durability and maintenance limitations [5]. Modern construction methods, while structurally superior, rely predominantly on Portland cement and natural aggregates. The rising cost of cement—often the most expensive component of concrete—combined with its environmental footprint, necessitates the exploration of alternative and locally available materials capable of partially replacing conventional constituents without compromising structural performance [6].

The incorporation of industrial and agricultural by-products as supplementary cementitious materials or aggregate substitutes has emerged as a promising strategy to reduce clinker consumption and promote circular economy principles. Materials such as fly ash, silica fume, steel slag, and agricultural ashes have demonstrated potential in improving mechanical and durability performance while reducing environmental impact [7,8,9,10,11,12]. Amin et al. [13] investigated the fresh and hardened properties of high-strength concrete (HSC) incorporating sugarcane bagasse ash (SCBA) and nano eggshell powder (NEP). The pozzolanic reaction of fly ash and untreated SCBA was found to reduce concrete porosity and enhance long-term compressive strength [14]. Alkali-activated mortars containing blast furnace slag (BFS) and sugarcane bagasse ash (SBA) exhibited superior durability performance compared with ordinary Portland cement mortars when exposed to acidic and sulphate environments [15]. Due to its pozzolanic activity, bagasse ash improved the hydration characteristics and microstructural refinement of cement pastes, while also enhancing the immobilization efficiency of Cr(VI) ions [16]. Furthermore, bagasse ash and black rice husk ash demonstrated considerable potential as sustainable sand replacement materials in the production of autoclaved aerated concrete [17].

Processed bagasse ash with high fineness and enhanced pozzolanic activity significantly influenced the properties of cement paste and mortar at replacement levels of up to 10% [18]. Agricultural waste materials, including wood bottom ash and banana fibers, have also shown significant potential for producing sustainable and thermally efficient cement-based composites [19]. Cement mortars incorporating 10% processed sugarcane bagasse ash exhibited improved mechanical and fracture properties, which were attributed to enhanced hydration and microstructural development [20]. Similarly, corn stover ash calcined at 600 °C demonstrated considerable potential as a partial slag replacement in alkali-activated slag systems due to its high pozzolanic activity and beneficial effects on hydration and strength development [21].

The use of chemical activators such as calcium formate and sodium metasilicate was shown to enhance hydration and early-age strength development in blast furnace slag blended cement systems, with calcium formate providing additional long-term benefits through improved slag reactivity [22]. Likewise, the incorporation of 10% sugarcane bagasse ash and 10% silica fume improved the mechanical performance of cement mortars owing to enhanced pozzolanic reactions and microstructural refinement [23]. The inclusion of up to 10% sugarcane bagasse ash also improved the fresh and mechanical properties of lightweight concrete while reducing embodied CO_2_ emissions [24]. In addition, sugarcane bagasse ash and corn stalk ash demonstrated significant potential as sustainable supplementary cementitious materials for enhancing the strength and durability of ultra-high-strength concrete [25].

Beyond agricultural residues, biosolid-derived materials such as biochar and bioash have shown promising potential as sustainable cement replacement materials due to their influence on porosity and mechanical performance [26]. Optimized seawater and sea-sand geopolymer concrete mixtures incorporating fly ash and slag exhibited improved workability and mechanical properties as a result of enhanced C-(A)-S-H gel formation [27]. Furthermore, FRP confinement significantly improved the bond behaviour and durability of epoxy-coated reinforcement embedded in seawater and sea-sand geopolymer concrete [28]. Similarly, seawater-sea sand geopolymer mortar demonstrated promising mechanical and environmental performance through optimized alkali activation and the incorporation of fly ash [29].

Among these alternatives, sugarcane bagasse ash (SCBA) and steel slag (SS) present significant opportunities within the Ethiopian context. Ethiopia possesses an expanding sugar industry with 13 factories and large-scale sugarcane plantations. The combustion of bagasse for energy generation produces substantial quantities of SCBA, estimated to reach nearly 2 million tons annually following full operational capacity [30]. Despite this availability, SCBA remains largely underutilized and is commonly disposed of as waste.

Similarly, steel slag, a by-product of steel manufacturing, is generated in considerable quantities by domestic steel industries in Ethiopia. Although national-level data on steel slag generation are limited, plant-based reports indicate that a single steel factory produces approximately 1.5–3 tons of slag per day, corresponding to about 550–1100 tons annually. Generally, steel slag accounts for approximately 15–20% of total steel production. Considering the increasing number of steel industries in the country, the total annual generation of steel slag is estimated to reach several thousand tons. This highlights the significant potential for sustainable reuse of steel slag in engineering and construction applications. Large volumes are stockpiled or landfilled, posing environmental and land-use challenges [31]. Steel slag possesses favorable physical and mechanical properties, including high density and angularity, which may enhance concrete performance when used as aggregate replacement. SCBA, depending on its chemical composition and fineness, can exhibit pozzolanic activity suitable for partial cement replacement.

The valorization of these industrial residues offers a dual benefit: mitigation of waste disposal challenges and reduction in reliance on Portland cement and natural sand. However, systematic evaluation of their combined use in concrete, particularly under Ethiopian material and production conditions, remains limited.

Therefore, this study investigates the feasibility of incorporating sugarcane bagasse ash as partial cement replacement and steel slag as both cement and sand replacement in concrete production. The research aims to identify optimal substitution levels that maintain structural-grade mechanical performance while enhancing sustainability. The physical and chemical characteristics of the materials are analyzed, and the effects of varying replacement ratios on fresh and hardened concrete properties are experimentally evaluated. The findings contribute to the development of resource-efficient and locally adaptable concrete mixtures suitable for sustainable construction practices in Ethiopia and similar developing contexts.

## 2. Materials and Methods

### 2.1. Raw Materials

**Figure 1:**
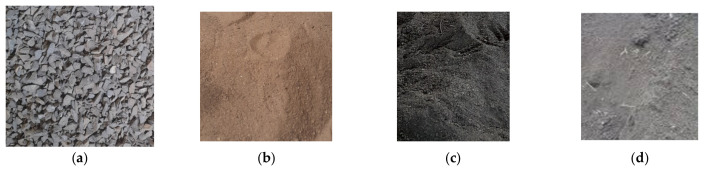
Raw materials used in the concrete mixes. (**a**) Crushed Stone (coarse aggregate); (**b**) River Sand (fine aggregate); (**c**) Steel Slag (fine aggregate and binder); (**d**) Sugarcane Bagasse Ash (binder).

The aggregates used in this study consisted of crushed stone, river sand, and steel slag (Figure 1a–c). Ordinary Portland Cement (CEM I 42.5N) was obtained from the Dangote Cement factory located in the West Shoa Zone, Oromia, Ethiopia. Sugarcane bagasse ash (SCBA) was collected from the Wonji Sugar Factory in the Oromia Regional State, Ethiopia. The bagasse ash (Figure 1d) was sun-dried for five days to remove residual moisture, followed by grinding to achieve finer particle size. The ground ash was then sieved through a 75 µm mesh to obtain fineness comparable to that of Portland cement. Steel slag was obtained from the Steely Rolling Mill Industry, Bishoftu, Oromia, Ethiopia. The slag was also sun-dried for five days to eliminate excess moisture, crushed, and sieved through a 75 µm mesh size to match the fineness of cementitious materials. Natural coarse aggregate and river sand were sourced from local construction material suppliers in Jimma City, Oromia, Ethiopia.

In this study, steel slag was utilized with a dual-purpose approach to maximize the valorization of this waste material. The fine fraction passing through the 75 μm sieve was used as a cementitious material (cement replacement), while the coarser fraction was used as fine aggregate (sand replacement). This combined strategy was adopted to ensure the comprehensive utilization of steel slag across different size fractions, thereby reducing construction waste and promoting sustainable material use in concrete production.

The chemical composition of cement, SCBA, and steel slag used for partial cement and sand replacement was determined at the Ethiopian Geological Survey Laboratory, and the results are presented in Table 1.

Crushed stone with a maximum nominal size of 37.5 mm was used as coarse aggregate. The coarse aggregate was sun-dried for five days prior to mixing to minimize moisture variability. Steel slag used as aggregate replacement was crushed and sieved to a maximum particle size of 37.5 mm. The selection of coarse aggregate with a maximum size of 37.5 mm was governed by standard specifications and performance requirements for normal concrete. In this study, the aggregate grading was designed in accordance with ASTM C33/C33M-18 [32] standard, which define acceptable grading limits for normal-weight concrete. Sieve analysis confirmed a maximum aggregate size of 37.5 mm (3.75 cm) and a nominal maximum size of 25 mm, both within the recommended range for structural concrete applications. This grading ensured adequate workability, proper compaction, and uniform particle distribution within the concrete matrix, contributing to improved homogeneity and overall performance. River sand was collected from a river source near Jimma, Ethiopia. The sand was tested to ensure that silt content remained below 5%, in accordance with ASTM C33 [32] requirements. Additionally, sieve analysis was performed to ensure a fineness modulus ranging between 2.3 and 3.1.

**Figure 2:**
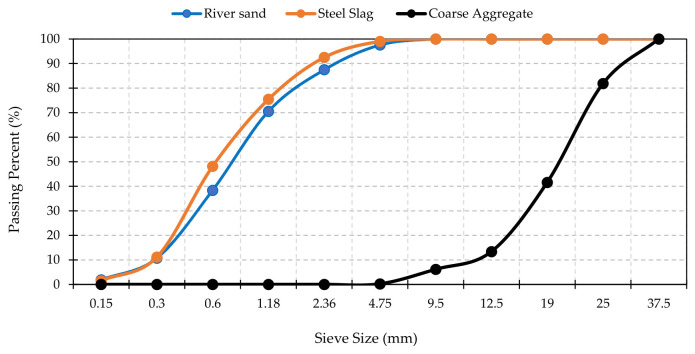
Particle size distribution of sand, steel slag and coarse aggregate.

The physical properties of river sand, coarse aggregate, and steel slag were evaluated following ASTM C136 [33] standards, and the results are summarized in Table 2. The particle size distributions of steel slag, river sand, and coarse aggregate are illustrated in Figure 2.

### 2.2. Experimental Plan

Concrete mixtures corresponding to M25 grade concrete were prepared as shown in Table 3 and Table 4. Three experimental programs were conducted to evaluate the effects of SCBA and steel slag as partial replacements for cement and natural sand. The reference mixture consisted of 370 kg/m^3^ of cement, 40% river sand, and 60% crushed granite aggregate.

In the first experimental stage, cement was partially replaced with bagasse ash cement (BAC) and steel slag cement (SSC) at replacement levels of 10%, 20%, and 30%, while river sand was partially substituted with steel slag sand (SSS) at the same percentages. The compressive strength and splitting tensile strength of the resulting concrete mixtures were evaluated to determine the influence of each replacement material on concrete performance and to establish acceptable substitution ranges.

Based on the findings of the initial evaluation, the second experimental stage focused on replacement levels that exhibited the most favorable mechanical performance while satisfying the target strength requirements. Consequently, 15% BAC and 25% SSC were selected as the optimum cement replacement levels, whereas 50% SSS was selected as the optimum sand replacement level due to its superior performance and greater replacement potential.

In the third experimental stage, binary and ternary blended systems were investigated using the selected replacement levels. The mixtures evaluated included BAC15% + SSC25%, BAC15% + SSS50%, SSC25% + SSS50%, and the ternary combination BAC15% + SSC25% + SSS50%. These mixtures were assessed to examine the combined effects of bagasse ash and steel slag on the mechanical performance of concrete and to identify the most suitable blend for structural applications.

The experimental results indicated that the BAC15% + SSS50% mixture exhibited the most favourable overall performance and was therefore selected for the subsequent structural concrete investigation.

A constant water-to-binder ratio of 0.50 was maintained for all mixtures. An overview of the conducted experimental program is given in Table 5.

### 2.3. Preparation of Test Specimens: Mixing, Casting and Curing

Concrete mixing was performed using a three-stage procedure. First, cement, SCBA, steel slag, and aggregates were dry-mixed for approximately three minutes to ensure uniform distribution. Second, half of the mixing water was added gradually and blended for two minutes. Finally, the remaining water was introduced and mixed for an additional three minutes to obtain a homogeneous mixture.

The fresh concrete mixture was then cast into steel molds and compacted using a steel rod to eliminate entrapped air. The specimens were cured in clean water at room temperature for 7 and 28 days. After curing, the specimens were removed from water, surface dried, and prepared for mechanical testing.

### 2.4. Test Methods

#### 2.4.1. Workability

The workability of fresh concrete was evaluated using the slump test in accordance with ASTM International standard ASTM C143 [34].

Fresh concrete was placed into a standard slump cone in three equal layers. Each layer was compacted using 25 strokes of a tamping rod to minimize air voids. After lifting the cone vertically, the reduction in height of the concrete was measured and recorded as the slump value.

#### 2.4.2. Compressive Strength

Compressive strength testing was conducted following ASTM C39 [35] procedures.

Concrete specimens were cast into 150 mm cube molds. After demolding, the specimens were immersed in curing water for 24 h before being transferred to curing tanks for 7-day and 28-day strength evaluation.

Compressive load was applied axially at a loading rate within the recommended range until specimen failure occurred. Compressive strength was calculated according to Equation (1).

(1) $$f_{c}=\frac{F}{A}$$

where *f_c_* is the concrete compressive strength (N/m^2^), *F* is the maximum load applied during the test (N) and *A* is the loaded area of the test specimen (mm^2^).

#### 2.4.3. Splitting Tensile Strength

Splitting tensile strength was determined using cylindrical specimens measuring 100 mm × 200 mm in accordance with ASTM C496 [36].

A diametral compressive load was applied along the longitudinal axis of the cylinder at a controlled loading rate until failure occurred. Splitting tensile strength was calculated according to Equation (2).

(2) $$f_{sp}=\frac{2F}{\pi DL}$$

where *f_sp_* is the splitting tensile strength (N/m^2^), *F* is the highest applied load that the testing apparatus indicates (N), *D* is the test specimen diameter in (mm) and *L* is the test specimen length (mm).

## 3. Results

### 3.1. Workability

**Figure 3:**
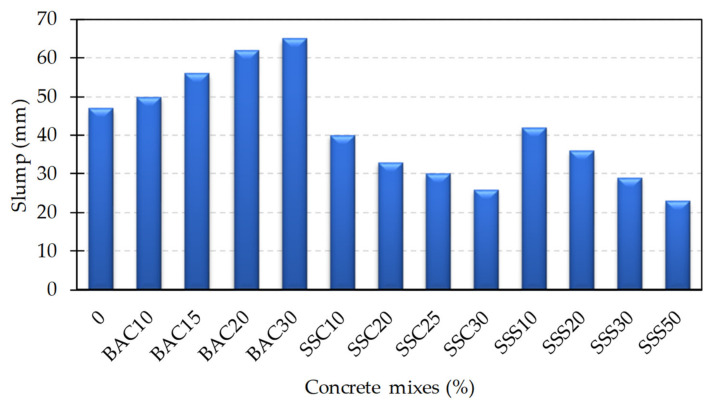
Slump values of concrete mixes with BAC and SS used as partial cement and sand replacements.

The workability of reference and modified concrete mixtures was evaluated using the slump test in accordance with ASTM C143 [34]. All mixtures were prepared and tested under identical conditions to ensure consistent comparison of fresh concrete behavior. The effects of SCBA and steel slag on slump are presented in Figure 3.

#### 3.1.1. Effect of Steel Slag

A progressive reduction in slump was observed with increasing replacement of natural sand and cement by steel slag. When steel slag was used as partial sand replacement, slump values decreased to 42, 36, 29, and 23 mm for replacement levels of 10%, 20%, 30%, and 50%, respectively. These values correspond to workability reductions of 14.3%, 26.5%, 40.8%, and 53.1% relative to the reference mixture.

Similarly, when steel slag was used as cement replacement, slump values of 40, 33, 30, and 26 mm were recorded for 10%, 15%, 20%, and 30% substitution levels, representing reductions of 18.4%, 32.7%, 38.8%, and 46.9%, respectively.

The reduction in workability is primarily attributed to the physical characteristics of steel slag. Compared to conventional aggregates and Ordinary Portland Cement, steel slag particles exhibit higher angularity, rough surface texture, and greater water absorption capacity. These properties increase internal friction within the fresh concrete matrix and reduce the amount of free water available for lubrication, thereby decreasing flowability. Similar trends have been reported in previous studies [37].

At higher replacement levels, the available mixing water becomes insufficient to ensure adequate particle mobility, resulting in stiffer mixtures.

#### 3.1.2. Effect of Sugarcane Bagasse Ash

In contrast, the incorporation of sugarcane bagasse ash (SCBA) as partial cement replacement enhanced the workability of concrete. Slump values of 50, 56, 62, and 65 mm were recorded for SCBA replacement levels of 10%, 15%, 20%, and 30%, respectively. Compared to the reference mixture, these values represent increases of 2.0%, 14.3%, 26.5%, and 32.7%.

The improved workability can be attributed to the finer particle size of SCBA and its micro-filler effect. The fine particles enhance particle packing and reduce interparticle friction, thereby improving paste lubrication within the cementitious matrix. As a result, lower water demand is required to achieve comparable flowability. This improvement is in good agreement with previous studies, which reported that bagasse ash contributes to enhanced flowability due to its fine particle size and microfiller effect [38].

Since all mixtures were prepared using a constant water-to-binder ratio of 0.50, the observed variations in slump are primarily governed by the intrinsic physical characteristics of the replacement materials rather than differences in water content.

### 3.2. Unit Weight of Concrete

Prior to compressive and splitting tensile strength testing, the dimensions and unit weight of all specimens were measured at 7 and 28 days of curing. The measured unit weights for all mixtures are presented in Table 6.

An increase in unit weight was observed with curing age for all mixes. This trend can be attributed to the continued hydration of cementitious materials, which leads to progressive densification of the concrete matrix. As hydration products accumulate and fill internal voids, the overall density of the concrete increases, resulting in slightly higher unit weight values at 28 days compared to 7 days.

For the reference mixture, the unit weight increased from 2434.30 kg/m^3^ at 7 days to 2442.96 kg/m^3^ at 28 days. A similar trend was observed for mixtures containing steel slag. For instance, concrete incorporating 50% steel slag as a sand replacement exhibited a unit weight of 2652.35 kg/m^3^ at 7 days, increasing to 2680.49 kg/m^3^ at 28 days.

The higher unit weight observed in steel slag-modified concrete is primarily associated with the greater specific gravity and density of steel slag compared to natural sand. Consequently, increasing steel slag content results in heavier concrete mixtures.

Overall, the results indicate that both curing age and steel slag incorporation influence concrete density, with steel slag contributing to increased unit weight due to its intrinsic material properties.

The unit weight of alternative concrete mixtures increased with increasing steel slag content, whether used as cement or sand replacement, and decreased with increasing bagasse ash (BAC) content as cement replacement (Table 6).

At 28 days of curing, the reference mixture exhibited a unit weight of 2442.96 kg/m^3^. In comparison, mixtures containing 30% BAC, 30% steel slag as cement replacement (SSC), and 50% steel slag as sand replacement (SSS) recorded unit weights of 2288.59 kg/m^3^, 2573.78 kg/m^3^, and 2680.49 kg/m^3^, respectively.

When steel slag was used as sand replacement at 10%, 20%, 30%, and 50%, the unit weight increased by 3.53%, 5.62%, 6.54%, and 9.72%, respectively, relative to the reference mixture. This increase is attributed to the higher bulk density and specific gravity of steel slag compared to natural sand, resulting in heavier concrete mixtures. A similar increasing trend was observed when steel slag was used as cement replacement.

Conversely, replacing cement with BAC resulted in a reduction in unit weight. At replacement levels of 10%, 15%, 20%, and 30%, decreases of 0.76%, 2.58%, 3.57%, and 6.32%, respectively, were observed. This reduction is primarily associated with the lower specific gravity of BAC (2.169 g/cm^3^) compared to Ordinary Portland Cement (3.15 g/cm^3^). The lower density of BAC reduces the overall mass per unit volume of the concrete mixture.

Although the reduction in unit weight due to BAC incorporation is moderate, lighter concrete offers potential structural benefits, including reduced dead load, smaller member sizes, and lower formwork pressure. Therefore, BAC incorporation may contribute not only to sustainability but also to improved structural efficiency.

### 3.3. Compressive Strength

**Figure 4:**
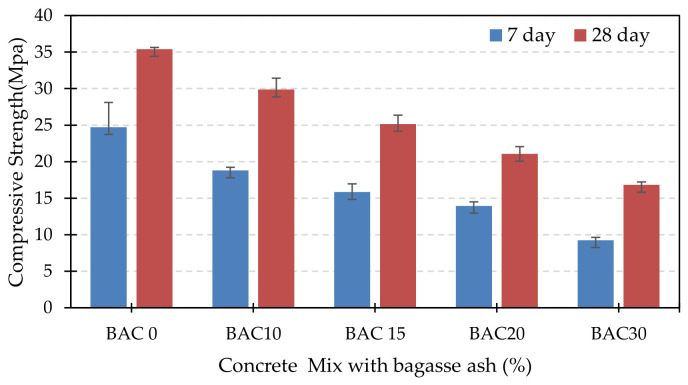
Compressive strength of reference and BAC concrete mixtures.

**Figure 5:**
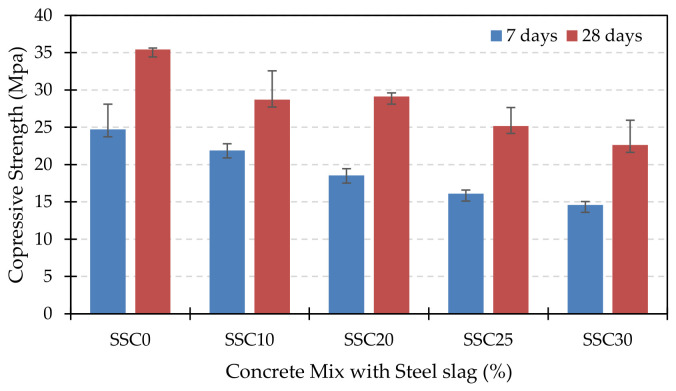
Compressive strength of reference and SSC concrete mixtures.

**Figure 6:**
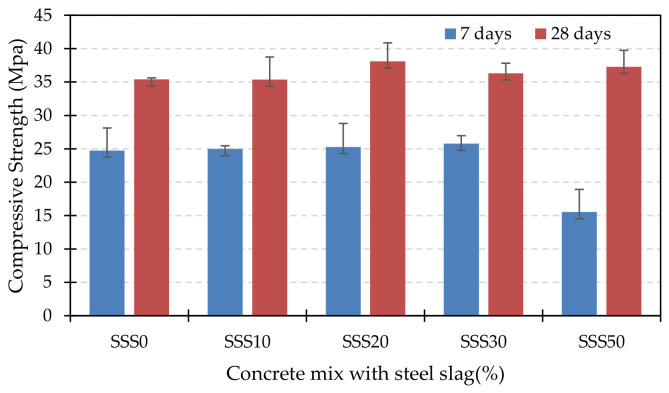
Compressive strength of reference and SSS concrete mixtures.

**Figure 7:**
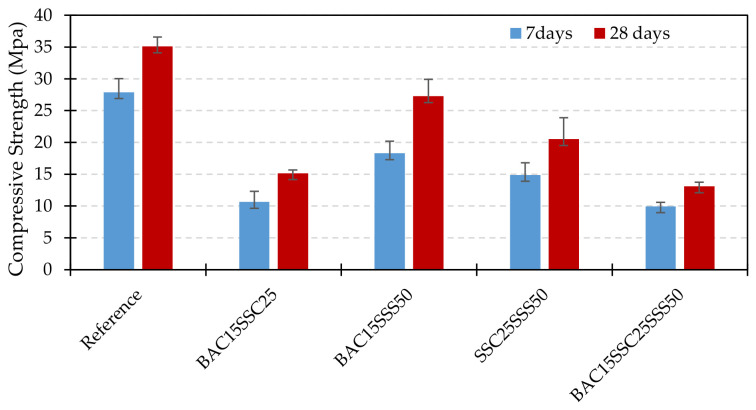
Compressive strength of reference and combined BAC–SSC–SSS concrete mixtures.

The compressive strength results of the reference and alternative concrete mixtures incorporating sugarcane bagasse ash (BAC) and steel slag (SSC and SSS) are presented in Figure 4, Figure 5, Figure 6 and Figure 7. The measured 28-day compressive strength values ranged from 13.09 MPa to 38.10 MPa, with most mixtures falling within the typical range of normal-strength concrete.

#### 3.3.1. Effect of Sugarcane Bagasse Ash (BAC)

Figure 4 illustrates the variation in compressive strength of the reference and BAC-modified concrete mixtures. A progressive decrease in compressive strength was observed with increasing BAC replacement levels. At 28 days, strength reductions of 15.65%, 28.97%, 40.54%, and 52.48% were recorded for BAC replacement levels of 10%, 15%, 20%, and 30%, respectively, relative to the reference mixture.

The observed reduction in compressive strength can primarily be attributed to the cement dilution effect and the relatively low early-age pozzolanic reactivity of BAC [39,40]. Replacing cement with BAC reduces the proportion of clinker phases (C_3_S and C_2_S), which are mainly responsible for hydration and early strength development. As a result, the formation of primary calcium silicate hydrate (C–S–H) gel is diminished, leading to lower compressive strength, particularly at higher replacement levels [40].

Despite this reduction, the reactive amorphous SiO_2_ contained in BAC may contribute to strength development through secondary pozzolanic reactions. During cement hydration, calcium hydroxide (Ca(OH)_2_) is generated as a by-product and can subsequently react with the reactive silica present in BAC to form additional secondary C–S–H gel. This process contributes to matrix densification and enhances bonding within the cementitious system [40,41]. The formation of secondary hydration products may also refine the pore structure, reduce microvoids, and improve the interfacial transition zone (ITZ) between the cement paste and aggregates [41].

In addition, the fine particle size of BAC may promote a micro-filler effect by filling voids between cement particles and improving particle packing density. This effect can reduce pore volume and contribute to a denser concrete microstructure [40,42]. At lower replacement levels, the combined effects of filler action and pozzolanic activity may partially compensate for the reduction in cement content and support strength development at later curing ages.

However, excessive BAC replacement levels adversely affect compressive strength because the amount of calcium hydroxide generated during cement hydration becomes insufficient to sustain effective secondary pozzolanic reactions. Moreover, the irregular particle morphology and porous structure of BAC may increase water demand, resulting in higher capillary porosity when a constant water-to-binder ratio is maintained [40,41]. The presence of unburnt carbon may further interfere with hydration processes and weaken the bond between the cement paste and aggregates. Consequently, the concrete matrix becomes less dense and more porous, leading to a reduction in load-carrying capacity.

Overall, the decrease in compressive strength with increasing BAC content can be attributed to four main factors: (i) reduced cementitious content resulting from dilution effects; (ii) delayed pozzolanic reactivity; (iii) increased porosity; and (iv) weakening of the interfacial transition zone (ITZ). Nevertheless, moderate BAC incorporation may still provide beneficial effects through secondary C–S–H formation and micro-filler action, particularly at later curing ages. These findings are in agreement with previous studies reported in the literature [39,40,41,42,43].

#### 3.3.2. Effect of Steel Slag as Cement Replacement (SSC)

The variation in compressive strength for reference and SSC-modified concrete is presented in Figure 5. The incorporation of steel slag as a partial replacement for cement resulted in a reduction in compressive strength, particularly at early curing ages. This behavior is mainly associated with the cement dilution effect and the lower hydraulic reactivity of steel slag compared to ordinary Portland cement.

Replacing cement with steel slag reduces the content of reactive clinker phases (C_3_S and C_2_S), thereby limiting the formation of primary calcium silicate hydrate (C–S–H) gel, which is the principal contributor to strength development. Unlike Portland cement, steel slag exhibits slower hydration kinetics, which delays strength gain, especially at early ages.

Furthermore, the presence of free CaO and MgO in steel slag may influence volumetric stability and microstructural development. The angular particle morphology and relatively rough surface texture can also increase internal friction and contribute to higher porosity if particle packing is not optimized. These factors may weaken the interfacial transition zone (ITZ) between paste and aggregate, thereby reducing load-transfer efficiency within the matrix.

As a result, mixtures incorporating steel slag as cement replacement generally exhibited lower compressive strength compared to the reference mix.

#### 3.3.3. Effect of Steel Slag as Sand Replacement (SSS)

The variation in compressive strength for mixtures incorporating steel slag as sand replacement is illustrated in Figure 6. The partial replacement of river sand with steel slag resulted in an improvement in 28-day compressive strength compared to the reference mixture. The compressive strength increased by 2.54%, 5.22%, and 7.57% at replacement levels of 20%, 30%, and 50%, respectively.

The observed enhancement in compressive strength can be attributed to both physical and limited chemical contributions of steel slag.

From a physical perspective, steel slag possesses higher hardness, angularity, and surface roughness compared to natural sand. These characteristics improve particle interlocking and mechanical bonding within the concrete matrix. The angular shape enhances aggregate interlock, while improved particle packing reduces internal voids, leading to a denser and more efficient stress-transfer mechanism under compressive loading.

In addition, steel slag contains reactive oxides such as CaO, SiO_2_, and Al_2_O_3_, which may contribute to secondary hydration reactions. These reactions can promote the formation of additional calcium silicate hydrate (C–S–H) gel, thereby enhancing the microstructure and strengthening the interfacial transition zone (ITZ) between aggregate and paste. However, the strength gain is primarily governed by improved aggregate–paste interaction rather than extensive hydraulic activity.

The results indicate that steel slag is particularly effective as a fine aggregate replacement, with optimal replacement levels observed between 20% and 50%, where compressive strength exceeded that of the reference mixture.

#### 3.3.4. Effect of Combined Replacement Systems (BAC + SSC + SSS)

Based on the final experimental phase, four combined replacement systems were evaluated: (i) BAC 15% + SSC 25%; (ii) BAC 15% + SSS 50%; (iii) SSC 25% + SSS 50%; (iv) BAC 15% + SSC 25% + SSS 50%. The compressive strength variations for combined replacement systems are presented in Figure 7. Among these combinations, only the BAC 15% + SSS 50% mixtures exhibited 28-day compressive strength values within the normal range for conventional structural concrete as established in EN 206-1 [44]. This mixture therefore satisfied the strength requirements for structural application and was considered suitable for the second phase of the study.

In contrast, mixtures incorporating combined cement replacement (BAC 15% + SSC 25% and SSC 25% + SSS 50%) showed a noticeable reduction in compressive strength. This reduction is primarily attributed to excessive cement dilution. The simultaneous substitution of cement with both bagasse ash and steel slag significantly reduces the effective clinker content available for hydration. While bagasse ash contributes delayed pozzolanic activity, its early-age reactivity is limited. Steel slag, although containing partially reactive oxides, does not fully compensate for the reduction in cementitious material. Consequently, the combined cement replacement (15% BAC + 25% SSC) results in insufficient formation of primary C–S–H gel and a less dense microstructure, leading to reduced compressive capacity. The triple replacement system (BAC 15% + SSC 25% + SSS 50%) further accentuated this dilution effect, as the reduction in binder content outweighed the mechanical benefits provided by steel slag as fine aggregate.

The results clearly indicate that strength performance in combined systems is strongly governed by the balance between binder reduction and aggregate enhancement. While steel slag as sand replacement (SSS 50%) improves compressive strength through improved particle packing and aggregate interlock, excessive cement substitution negatively affects the hydration process and matrix integrity.

#### 3.3.5. Failure Behaviour Under Compressive Loading (CS)

**Figure 8:**
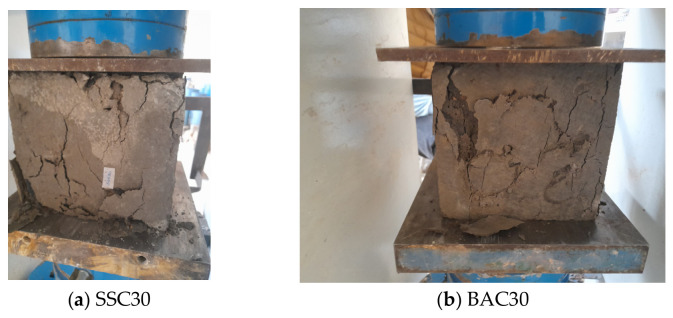
Illustration of observed compression test failure.

Under compressive loading, the reference concrete exhibited a relatively brittle failure behavior, characterized by rapid crack propagation and sudden failure immediately after reaching the peak load. The typical failure modes observed during the compression tests are presented in Figure 8. The reference specimens experienced pronounced crushing and fragmentation, indicating limited deformation capacity and low energy absorption.

In contrast, the concrete mixtures incorporating bagasse ash and steel slag exhibited a more gradual failure process, accompanied by reduced crack severity and less extensive fragmentation. These modified specimens retained greater structural integrity after peak loading, suggesting an improved resistance to crack initiation and propagation. This behavior may be attributed to the densification of the cementitious matrix and the enhancement of the interfacial transition zone (ITZ) resulting from the pozzolanic activity and micro-filler effect of the incorporated materials. These mechanisms contribute to a denser, more homogeneous, and cohesive microstructure, thereby improving the ability of the concrete to withstand compressive loading and delaying the progression of failure.

### 3.4. Splitting Tensile Strength

**Figure 9:**
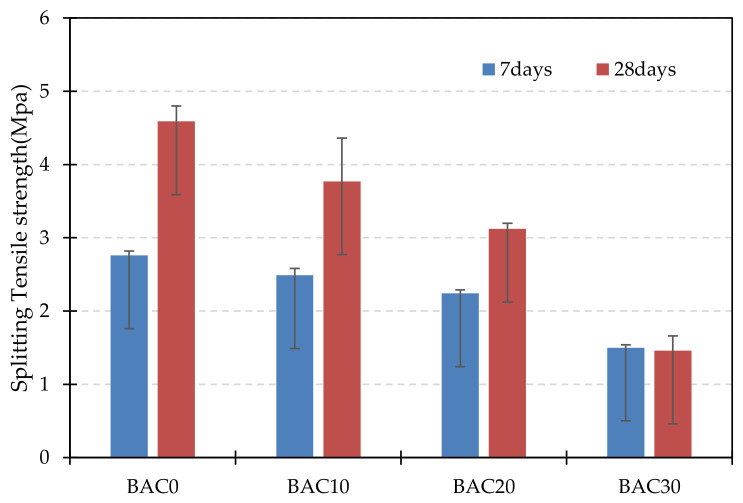
Splitting tensile strength of reference and BAC concrete mixtures.

**Figure 10:**
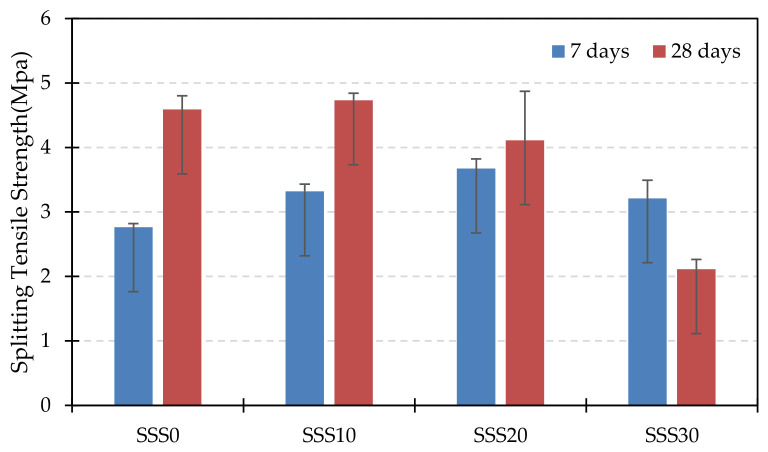
Splitting tensile strength of reference and SSS concrete mixtures.

**Figure 11:**
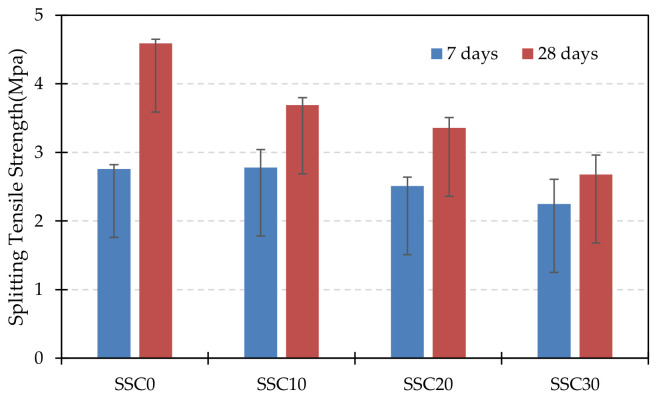
Splitting tensile strength of reference and SSC concrete mixtures.

**Figure 12:**
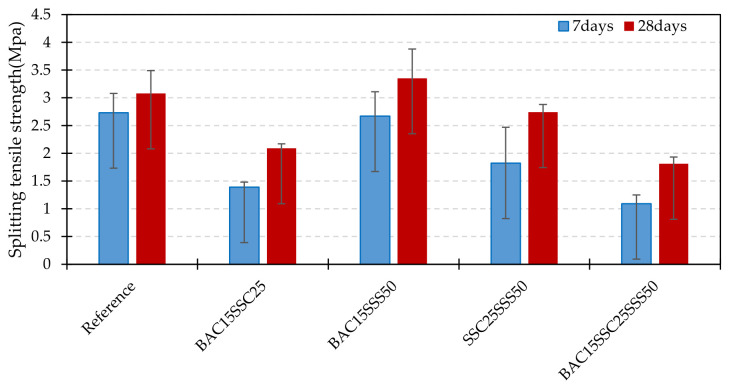
Splitting tensile strength of reference and alternative concrete mixtures at 7 and 28 days of curing.

The splitting tensile strength results of the reference and modified concrete mixtures are presented in Figure 9, Figure 10, Figure 11 and Figure 12. In general, the splitting tensile strength trends followed patterns similar to those observed for compressive strength, with reductions occurring in mixtures containing cement substitution and comparatively more stable behavior observed when steel slag was used as fine aggregate replacement.

#### 3.4.1. Effect of Bagasse Ash (BAC)

Concrete mixtures incorporating bagasse ash (BAC) as a partial cement replacement exhibited lower splitting tensile strength compared with the reference mixture. This reduction can primarily be attributed to the cement dilution effect, which decreases the amount of reactive clinker phases available for hydration. As a result, the formation of primary calcium silicate hydrate (C–S–H) gel, which plays a critical role in tensile stress transfer and crack resistance, is reduced [39,40].

However, the active amorphous SiO_2_ present in BAC may contribute to tensile strength development through secondary pozzolanic reactions. During cement hydration, calcium hydroxide (Ca(OH)_2_) is produced as a hydration by-product. The reactive silica contained in BAC can subsequently react with Ca(OH)_2_ to generate additional secondary C–S–H gel [40,41]. The formation of this secondary hydration product may improve matrix densification, refine the pore structure, and enhance the interfacial transition zone (ITZ) between the cement paste and aggregates. These improvements may increase stress transfer efficiency and enhance resistance to crack initiation and propagation under tensile loading [40,41].

Furthermore, the fine particles of BAC may provide a micro-filler effect by improving particle packing and reducing internal voids within the concrete matrix [40,42]. This filler action may contribute to improved bonding between the cement paste and aggregates, resulting in comparatively more gradual crack propagation. At lower BAC replacement levels, these pozzolanic and filler effects may partially compensate for the reduction in cementitious content and contribute to later-age tensile strength development.

Nevertheless, excessive BAC replacement levels may adversely affect tensile performance because the amount of calcium hydroxide generated during cement hydration becomes insufficient to sustain effective secondary pozzolanic reactions. In addition, the porous structure and irregular morphology of BAC particles may increase water demand and capillary porosity, thereby weakening the concrete microstructure [39,40]. The possible presence of unburnt carbon may further interfere with cement hydration and weaken the paste aggregate bond, resulting in reduced crack-bridging capacity within the cement matrix.

Therefore, the reduction in splitting tensile strength with increasing BAC content is mainly associated with: (i) reduced binder content due to dilution effects; (ii) increased porosity; (iii) weakened interfacial transition zones (ITZ); and (iv) reduced crack-bridging capacity within the cement matrix. However, moderate BAC incorporation may still provide beneficial effects through secondary C–S–H formation, pore refinement, and filler action, particularly at later curing ages.

The present interpretations are based on findings reported in previous studies [39,40,41,42,43]. Microstructural analyses such as scanning electron microscopy (SEM) and X-ray diffraction (XRD) were not conducted in the present study due to laboratory limitations. Therefore, future investigations are recommended to further evaluate hydration products, pore structure evolution, and ITZ development in BAC–SS blended concrete systems.

#### 3.4.2. Effect of Steel Slag as Sand Replacement (SSS)

When steel slag was used as partial sand replacement, the splitting tensile strength showed a slight reduction or marginal variation compared to the reference mixture. Although steel slag improves aggregate interlocking due to its angularity and rough surface texture, its higher stiffness and hardness may introduce localized stress concentrations within the matrix.

The angular particles can act as crack initiation points under tensile stress, increasing brittleness and facilitating crack propagation. As tensile strength is more sensitive to microcracking and interfacial defects than compressive strength, improvements observed in compressive performance with SSS were not fully reflected in tensile behavior.

#### 3.4.3. Effect of Steel Slag as Cement Replacement (SSC)

Partial replacement of cement with steel slag also resulted in reduced splitting tensile strength. Similar to compressive strength behavior, this reduction is linked to decreased availability of reactive clinker phases and the relatively slow hydration kinetics of steel slag.

The lower formation of C–S–H gel weakens the bond between paste and aggregates, reducing cohesion and tensile load-transfer efficiency. Furthermore, incomplete hydration may produce a less dense microstructure, increasing susceptibility to crack initiation and propagation under tensile loading.

#### 3.4.4. Effect of Combined Replacement Mixes (BAC + SSC + SSS)

The variation in splitting tensile strength at 7 and 28 days for the alternative mixtures is presented in Figure 11. Overall, the results confirm that tensile performance in combined mixes is governed by the balance between binder reduction and aggregate enhancement.

The results of the final testing phase indicate that mixtures incorporating bagasse ash as cement replacement combined with steel slag as sand replacement (BAC 15% + SSS 50%), as well as mixtures containing steel slag as both cement and sand replacements (SSC 25% + SSS 50%), achieved splitting tensile strength values within the normal range for structural concrete (2.5–4.5 MPa).

The splitting tensile strength trends were consistent with the compressive strength results. Among the four combined replacement mixes evaluated (BAC 15% + SSC 25%, BAC 15% + SSS 50%, SSC 25% + SSS 50%, and BAC 15% + SSC 25% + SSS 50%), only the combinations that incorporated steel slag as sand replacement (SSS 50%) satisfied the tensile strength requirements for structural applications.

The improved tensile performance in BAC 15% + SSS 50% mixture is primarily attributed to enhanced particle packing density and improved aggregate–paste interfacial bonding provided by steel slag sand. The angularity and surface roughness of steel slag promote stronger mechanical interlock and more efficient stress transfer under diametral loading. As tensile strength is highly sensitive to microcrack initiation and interfacial defects, improvements in the interfacial transition zone (ITZ) significantly contribute to crack resistance.

Conversely, mixtures with higher levels of combined cement replacement (BAC 15% + SSC 25%, SSC25%+ SSS50% and BAC 15% + SSC 25% + SSS 50%) exhibited reduced splitting tensile strength. This reduction is mainly associated with excessive cement dilution, which limits C–S–H formation and weakens paste–aggregate bonding. The resulting less-dense matrix facilitates earlier crack initiation and propagation under tensile stress.

#### 3.4.5. Failure Behaviour Under Splitting Tensile Loading (STS)

**Figure 13:**
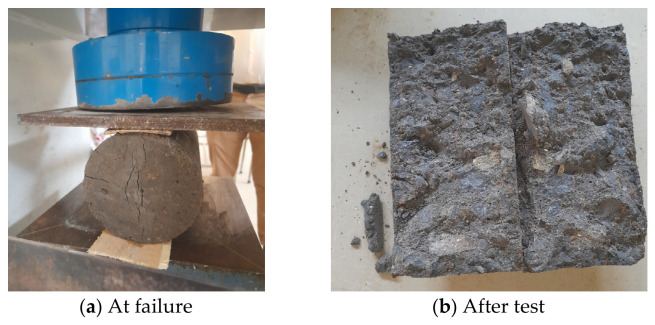
Illustration of observed splitting test failure.

During the splitting tensile tests, the reference concrete exhibited a typical brittle failure mode characterized by the formation of a pronounced longitudinal crack followed by rapid separation of the specimen (Figure 13). This behavior reflects the limited tensile deformation capacity of conventional concrete. In contrast, the concrete mixtures incorporating bagasse ash and steel slag exhibited a more gradual crack propagation process and retained partial structural integrity after crack initiation.

The reduced crack severity and delayed specimen separation suggest an improvement in crack resistance and energy absorption capacity. This enhanced tensile performance may be attributed to the densification of the cementitious matrix and the strengthening of the interfacial transition zone (ITZ) resulting from the pozzolanic activity and micro-filler effect of the supplementary materials. These mechanisms promote a more uniform stress distribution within the concrete, thereby delaying crack propagation and improving the overall tensile response of the composite material.

## 4. Comparative Analysis of Concrete Properties

### 4.1. Comparative Analysis of Workability

**Figure 14:**
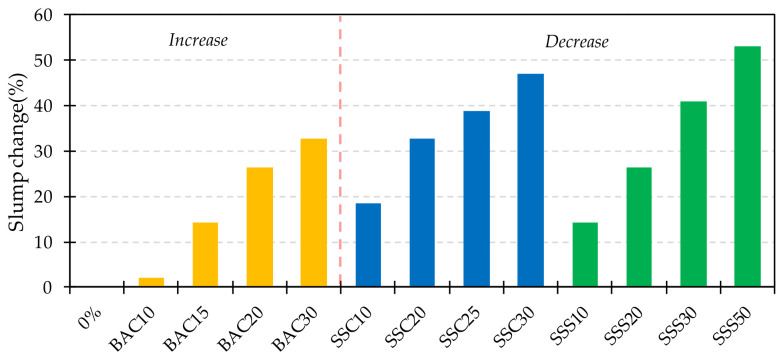
Slump variation of concrete mixtures with BAC (yellow), SSC (blue), and SSS (green) replacements relative to the reference concrete.

The influence of steel slag sand and sugarcane bagasse ash as sand and cement replacement on concrete workability is compared in Figure 14.

Steel slag incorporation resulted in a progressive reduction in slump value with increasing replacement levels, indicating decreased workability. This behavior is primarily associated with the angular particle morphology, higher surface roughness, and greater water absorption capacity of steel slag, which increase internal friction within the fresh concrete matrix.

In contrast, sugarcane bagasse ash replacement produced a gradual increase in slump values. The improvement in workability is attributed to the fine particle size and micro-filler effect of bagasse ash, which enhances particle packing and reduces interparticle friction. Under a constant water-to-binder ratio, the finer BAC particles improve paste lubrication and flowability.

The percentage change analysis demonstrates opposite behavioral trends for the two materials. Steel slag replacement reduced slump by up to 53.1%, whereas bagasse ash increased slump by up to 32.7% relative to the reference mixture.

These findings indicate that steel slag primarily influences fresh concrete rheology through physical aggregate characteristics, while bagasse ash affects workability through particle size distribution and packing density effects.

### 4.2. Comparative Analysis of Compressive Strength

**Figure 15:**
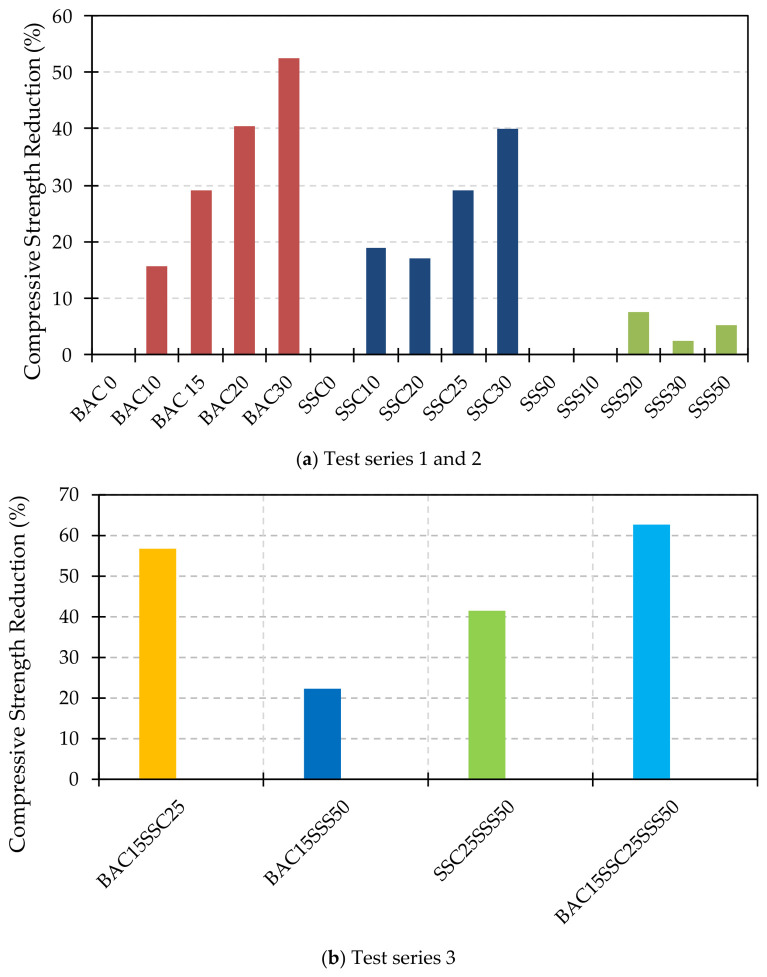
Compressive strength variation of concrete mixtures with BAC, SSC, and SSS replacements.

Figure 15 illustrates the compressive strength variation for BAC, SSC, and SSS replacement mixes. The compressive strength results indicate that partial replacement of conventional concrete constituents with industrial by-products can be achieved without significant strength loss up to specific substitution levels.

Concrete incorporating sugarcane bagasse ash (BAC) maintained compressive strength comparable to the reference mixture up to 15% replacement. Beyond this level, compressive strength decreased, which is primarily attributed to incomplete or delayed pozzolanic reactions and reduced cementitious material content. The limited early-age reactivity of BAC contributes to slower formation of calcium silicate hydrate (C–S–H) gel, resulting in reduced matrix densification at higher replacement ratios.

Concrete containing steel slag as cement replacement (SSC) exhibited comparable compressive strength to conventional concrete up to 25% substitution. This behavior suggests a partial hydraulic contribution of steel slag to the cementation process, although its reactivity is lower than that of Portland cement.

Notably, concrete mixtures incorporating steel slag as sand replacement (SSS) achieved compressive strength comparable to the reference mixture even at 50% replacement. This performance is primarily associated with the physical characteristics of steel slag, including higher hardness, angular particle shape, and improved aggregate interlocking, which enhance load transfer within the concrete matrix.

Overall, the results indicate that steel slag is more suitable as a fine aggregate substitute than as a binder replacement. The maximum sustainable replacement levels without significant strength reduction were observed at 15% BAC, 25% SSC, and 50% SSS for “single” replacements. For combined replacements only for 15%BAC + 50%SSS acceptable results were obtained. Strength degradation at higher replacement levels is mainly associated with excessive cement dilution and reduced hydration product formation.

## 5. Conclusions

The chemical composition of steel slag was assessed in relation to the ASTM C618 requirements for supplementary cementitious materials. The combined content of SiO_2_, Al_2_O_3_, and Fe_2_O_3_ provides an initial indication of potential pozzolanic behavior. However, this evaluation is limited to chemical criteria and does not represent full compliance with ASTM C618 specifications. According to ASTM C618, additional performance-based tests, such as the strength activity index, are required to confirm pozzolanic activity. Therefore, the results suggest that steel slag may exhibit potential pozzolanic characteristics when used as a partial cement replacement, rather than confirming complete pozzolanic classification.

This study investigated the chemical composition and mechanical performance of concrete incorporating sugarcane bagasse ash (BAC) and steel slag (SS) as partial replacements for cement and natural sand. The combined chemical composition of SiO_2_, Al_2_O_3_, and Fe_2_O_3_ in both BAC and SS satisfied the pozzolanic material requirements specified in ASTM C618 [5], indicating their potential suitability as supplementary cementitious materials.

The results showed that unit weight of concrete increased with increasing steel slag content due to the higher density and specific gravity of steel slag compared to natural sand. Conversely, incorporation of bagasse ash reduced unit weight because of its lower material density.

Optimal mechanical performance was achieved at 15% BAC cement replacement, 25% SSC cement replacement, and 50% SSS sand replacement. In combined replacement mixes, mixtures containing BAC 15% + SSS 50% exhibited compressive and splitting tensile strength values within the range required for structural concrete applications.

Increasing bagasse ash replacement beyond 15% resulted in compressive strength reduction due to cement dilution, delayed pozzolanic reactivity, increased porosity, and weaker interfacial transition zone development. Similarly, excessive steel slag cement replacement reduced strength because of lower availability of reactive clinker phases and slower hydration kinetics.

Splitting tensile strength exhibited trends consistent with compressive strength behavior, with values ranging between 1.81 MPa and 4.74 MPa at 28 days of curing.

The findings of this study provide experimental evidence supporting the sustainable utilization of agricultural and industrial by-products in concrete production. The incorporation of sugarcane bagasse ash and steel slag offers a promising pathway toward reducing cement and natural river sand consumption, minimizing construction-related environmental impacts, and promoting circular material use in the construction industry. Future research may focus on long-term durability performance, microstructural characterization, and life cycle environmental assessment of alternative concrete mixes. Optimization of mix design parameters and investigation of performance under aggressive environmental conditions are also recommended to further validate the structural applicability of these materials in large-scale construction projects.

## Figures and Tables

**Table 1:** Chemical composition of cement, bagasse ash and steel slag.

Comp (%)	SiO_2_	Al_2_O_3_	Fe_2_O_3_	CaO	MgO	Na_2_O	K_2_O	MnO	P_2_O_5_	TiO_3_	H_2_O	LOI	SiO_2_ + Al_2_O_3_ + Fe_2_O_3_
OPC	22.82	5.41	3.37	66.32	1.46	_	_	_	_	_	_	_	31.6
SCBA	61.04	6.12	3.52	1.36	2.56	1.08	5.92	0.01	0.66	0.28	1.88	10.15	72.68
SS	55.16	15.40	13.08	2.62	1.30	0.62	<0.01	11.20	0.08	0.47	0.20	<0.01	83.64

**Table 2:** Key physical properties of aggregates used in this study.

Physical Properties	RS	SS	CA
Silt content (%)	1.29	1.10	0
Fineness modulus (%)	2.93	2.723	4.6
Bulk Specific gravity (SSD)	2.62	4.90	2.69
Water absorption capacity (%)	1.18	0.23	0.44
Maximum coarse aggregate size (mm)	4.75	4.75	37.5

**Table 3:** Mix design calculation per cubic meter of normal concrete.

Ingredients	Cement	Sand	CA	Water
Quantity (Kg/m^3^)	370	715.6	1100	192
Ratio	1	1.93	2.97	0.52
Quantity in Kg	50	97	149	25.9

**Table 4:** Mix design calculation per cubic meter of alternative concrete.

Ingredients	Cement	BAC	SSC	Sand	SSS	CA	Water
Quantity (Kg/m^3^)	370	276.5	313.5	715.6	787.16	1100	192
Ratio	1	1	1	1.93	1.93	2.97	0.52
Quantity in Kg	50			97		149	25.9

**Table 5:** The specimen type and number of tests.

Test Series	Specimens	Compressive		Splitting		Total per Specimen Type	Total per Test Series
		Cube 150 mm^3^		Cylinder D = 100 mm L = 200 m			
		7 Days	28 Days	7 Days	28 Days		
1	Ref; BAC10%; BAC20%; BAC30%; SSC10%; SSC20%; SSC30%; SSS10%; SSS20%; SSS30%	3	3	3	3	12	120
2	BAC15%; SSC25%; SSS50%	3	3	-	-	6	18
3	BAC15%SSC25%; BAC15%SSS50%; SSC25%SSS50%; BAC15%SSC25%SSS50%	3	3	3	3	12	48
						Total	186

**Table 6:** Unit weight of reference and alternative concrete mixtures.

Composition (%)	Compressive Strength Specimens Unit Weight (Kg/m^3^)		Variation (%) 28 Days	Splitting Tensile Strength Specimens Unit Weight (Kg/m^3^)		Variation (%) 28 Days
	7 Days	28 Days		7 Days	28 Days	
BAC0	2434.30	2442.96	0	2369	2760	0
BAC10	2417.20	2424.30	−0.76	2351	2347	−14.96
BAC15	2325.58	2379.90	−2.58	_	_	
BAC20	2338.70	2355.85	−3.57	2305	2298	−16.74
BAC30	2282.07	2288.59	−6.32	2329	2256	−18.26
SSC0	2434.30	2442.96	0	2369	2760	0
SSC10	2484.15	2496.89	+2.21	2326	2390	−13.41
SSC20	2503.70	2536.30	+3.82	2422	2441	−11.56
SSC25	2533.14	2563.16	+4.92	_	_	
SSC30	2504.59	2573.78	+5.35	2468	2481	−10.11
SSS0	2434.30	2442.96	0	2369	2760	0
SSS10	2472.29	2529.19	+3.53	2452	2475	−10.33
SSS20	2545.19	2580.15	+5.62	2484	2485	−9.96
SSS30	2560.89	2602.67	+6.54	2508	2589	−6.20
SSS50	2652.35	2680.49	+9.72	_	_	
BAC0SSC0SSS0	2434.30	2550.62	0	2369	2760	0
BAC15SSC25	2590.52	2424.89	+4.93	2361	2392	−13.33
BAC15SSS50	2898.17	2787.16	+9.27	2673	2710	−1.81
SSC25SSS50	2862.22	2781.83	+9.06	2605	2674	−3.12
BAC15SSC25SSS50	2900.25	2785.48	+9.21	2706	2793	−1.2

## Data Availability

The original contributions presented in this study are included in the article. Further inquiries can be directed to the corresponding author.

## Abbreviations

The following abbreviations are used in this manuscript:

BA	Bagasse Ash
BAC	Bagasse Asha as Cement replacement
SS	Steel Slag
SSC	Steel Slag as Cement replacement
SSS	Steel Slag as Sand replacement
OPC	Ordinary Portland Cement
CS	Compressive Strength
STS	Splitting Tensile Strength
RS	River Sand
CA	Coarse Aggregate
